# Flooding and Emergency Room Visits for Gastrointestinal Illness in Massachusetts: A Case-Crossover Study

**DOI:** 10.1371/journal.pone.0110474

**Published:** 2014-10-17

**Authors:** Timothy J. Wade, Cynthia J. Lin, Jyotsna S. Jagai, Elizabeth D. Hilborn

**Affiliations:** 1 United States Environmental Protection Agency, National Health and Environmental Effects Research Laboratory, Environmental Public Health Division, Chapel Hill, North Carolina, United States of America; 2 Oak Ridge Institute for Science and Education (ORISE) Research Participation Program at the United States Environmental Protection Agency, Chapel Hill, North Carolina, United States of America; 3 University of North Carolina, Gillings School of Global Public Health, Department of Epidemiology, Chapel Hill, North Carolina, United States of America; 4 University of Illinois, Chicago, School of Public Health, Division of Environmental and Occupational Health Sciences, Chicago, Illinois, United States of America; Kliniken der Stadt Köln gGmbH, Germany

## Abstract

**Introduction:**

Floods and other severe weather events are anticipated to increase as a result of global climate change. Floods can lead to outbreaks of gastroenteritis and other infectious diseases due to disruption of sewage and water infrastructure and impacts on sanitation and hygiene. Floods have also been indirectly associated with outbreaks through population displacement and crowding.

**Methods:**

We conducted a case-crossover study to investigate the association between flooding and emergency room visits for gastrointestinal illness (ER-GI) in Massachusetts for the years 2003 through 2007. We obtained ER-GI visits from the State of Massachusetts and records of floods from the National Oceanic and Atmospheric Association’s Storm Events Database. ER-GI visits were considered exposed if a flood occurred in the town of residence within three hazard periods of the visit: 0–4 days; 5–9 days; and 10–14 days. A time-stratified bi-directional design was used for control selection, matching on day of the week with two weeks lead or lag time from the ER-GI visit. Fixed effect logistic regression models were used to estimate the risk of ER-GI visits following the flood.

**Results and Conclusions:**

A total of 270,457 ER-GI visits and 129 floods occurred in Massachusetts over the study period. Across all counties, flooding was associated with an increased risk for ER-GI in the 0–4 day period after flooding (Odds Ratio: 1.08; 95% Confidence Interval: 1.03–1.12); but not the 5–9 days (Odds Ratio: 0.995; 95% Confidence Interval: 0.955–1.04) or the 10–14 days after (Odds Ratio: 0.966, 95% Confidence Interval: 0.927–1.01). Similar results were observed for different definitions of ER-GI. The effect differed across counties, suggesting local differences in the risk and impact of flooding. Statewide, across the study period, an estimated 7% of ER-GI visits in the 0–4 days after a flood event were attributable to flooding.

## Introduction

Floods are the most common type of natural disaster and are responsible for numerous adverse acute and chronic health effects ranging from vector-borne and waterborne infections to injury and drowning [1]. Climate change could bring changes in the patterns of severe weather events and potential increases in precipitation, flooding and hurricanes [2]. Flooding has also been associated with increased greenhouse gas emissions [3].

Flooding is a well-documented risk factor for the transmission of infectious diseases [4]. Flooding can result in the discharge of untreated sewage and other wastes that could contaminate land, water supplies, watersheds or crops. Floods can increase human contact with fecal contamination through disruption of access to potable water, bypasses in sewage treatment impacting water quality, direct contact with sewage contaminated flood water, damages to water infrastructure compromising water treatment and through contact with contaminated food, surfaces and materials. In addition to direct or indirect waterborne and foodborne transmission of infection, floods can result in displacement of populations from their normal places of residence and congregation in shelters, resulting in crowding and increased person-to-person contact. There is further potential for substandard hygienic conditions resulting from power outages and contamination of food sources. Outbreaks of diarrhea are common following floods, most notably in underdeveloped regions, often caused by waterborne pathogens, such as *Vibrio cholerae* [5]. Other examples of potentially waterborne infections associated with flooding include typhoid and paratyphoid fever, cholera, hepatitis A, leptospirosis, shigellosis, campylobacteriosis, amebiasis, giardiasis, cryptosporidiosis, norovirus, and pathogenic *E. coli* [6].

In developed countries there is more limited evidence that floods may be a risk factor for the transmission of gastrointestinal infections. In both the United States and United Kingdom, precipitation events have been found to precede waterborne disease outbreaks [7], [8]. Increased diarrhea and gastrointestinal symptoms have been reported following a tropical storm [9], following flooding in the United States [10] and the United Kingdom [11] and among triathletes who swallowed water following a heavy rainfall [12]. Flooding of a hotel was associated with a norovirus outbreak among American tourists and firefighters in Vienna, Austria where the affected tourists helped staff clean water from the hotel sanitation system [13]. A recent cohort study found an increased risk of illness associated with floodwater contact among those who failed to wash their hands [14]. Urban flood waters in the Netherlands were found to be contaminated with potentially pathogenic microorganisms *Campylobacter jejuni*, *Giardia spp., Cryptosporidium spp.*, enteroviruses and noroviruses [15].

The goal of this study was to further explore the association between flooding and the risk for gastrointestinal illness in the United States using records of emergency room diagnoses for acute gastrointestinal illness and historical records of flooding in the state of Massachusetts.

## Methods

### Emergency Room Visits for Gastrointestinal Illness

Daily emergency room visits (ER visits) for acute gastrointestinal illness (GI) were obtained from the State of Massachusetts Division of Health Care Finance and Policy, Executive Office of Health and Human Services for the years 2003 through 2007 (these data are now compiled and maintained by the State of Massachusetts, Center for Health Information and Analysis). Reporting of outpatient emergency department records is mandated by Massachusetts state law. This database contains patient-level information, including socio-demographics, clinical data and discharge information. The hospitals report data to the Division on a quarterly basis and yearly databases are made available to the public through an application process. Information obtained included town and zip code of residence, discharge date, age, sex, primary diagnostic code (International Classification of Disease, Version 9 Clinical Modification (ICD-9CM)) and five associated diagnostic codes.

Massachusetts outpatient ER visits were subset based on ICD-9CM codes to include those diagnosed with acute gastrointestinal (ER-GI) illness. Cases which included the following ICD-9CM codes in the principal or one of five associated diagnostic codes were abstracted: 001-009, 558.9, 787, 787.0, 787.4, 787.9, 787.91. We excluded *Clostridium difficile* diagnoses (008.45) because it is the leading cause of infectious diarrhea in hospitalized patients and, although community-acquired cases appear to be increasing, it is still a predominantly hospital-acquired infection [16]. We also considered a second definition which included non-specific nausea with vomiting (787.01) and vomiting alone (787.03).

Because the data were acquired by state of Massachusetts for administrative purposes and were not obtained through intervention or interaction with any individual and because they do not contain identifiable private information they were completely anonymous and determined not to be data acquired from human subjects by the U.S. Environmental Protection Agency’s Human Subjects Research Protocol Officer. Thus informed consent was not needed and this research was considered exempt from Institutional Review Board review.

### Flood Events

Information on flood events was obtained from the Storm Events Database maintained and compiled by the National Oceanic & Atmospheric Administration (NOAA), National Weather Service (http://www.ncdc.noaa.gov/stormevents/). This database documents weather events that fall into the following categories: 1) storms and other significant weather events having intensity to cause loss of life, injuries, significant property damage and/or disruption to commerce; 2) rare or unusual weather phenomena that generate media attention; 3) other significant meteorological events such as record temperatures. Storms with the event type “Flood”, which included “Coastal Floods”, “Flash Floods”, and related events occurring in the State of Massachusetts for the time period December 2002 through January 2008 were included in the analysis. Storms from 2002 and 2008 were included to allow for lagged and leading exposures of the 2003 ER visits and control periods. The Storm Events Database included start and end dates of the flood, and counties and towns affected. Severe weather events from this database have been used previously in published peer reviewed manuscripts [17], [18].

### Study Design

We used a case-crossover study design to investigate the association between flood events impacting a specific town and ER-GI visits from residents of the flood-impacted town (see *Exposure Classification and Referent Periods* section below). In the case-crossover design, cases serve as their own control or referent at a different time period before or after the disease event. The case-crossover design is useful when studying transient exposures and acute effects because fixed individual characteristics (e.g., sex, race) that do not vary with time are controlled by design [19]. We hypothesized that the hazard period (defined as the time interval between flooding and elevated ER-GI visits [20]) in which floods most significantly impact the risk of ER-GI visits could range from 0 to 14 days following flooding depending on the route of exposure (e.g., direct contact with flood waters or flood contaminated items, or via contaminated water or food) and the incubation period of the infectious organism. While delayed effects of greater than 14 days are possible (*e.g.,* gastrointestinal symptoms from hepatitis A, or delayed effects from contaminated food, drinking water exposures, and secondary contact with those previously infected) these are likely to be less frequent and the association with flooding may be sporadic. We anticipated that most cases of acute GI illness attributable to flood exposure would occur soon after the flood. In order to gain insight on the time period between flooding and increased risk, we divided hazard periods into mutually exclusive time windows of 0–4, 5–9 and 10–14 days following the flood event. We hypothesized that effects in the first 0–4 days following the flood would likely be due to direct contact with flood waters and/or infections with short incubation periods (*e.g.,* enteric viruses), whereas as 5–9 days and 10–14 days could represent indirect exposure (*e.g.,* through drinking water, contaminated food, population displacement) or infections with longer incubation periods.

### Exposure Classification and Referent Periods

Whenever possible, town was used to define exposure. In other words, only specific towns impacted by the flood were considered exposed and other towns within the same county were considered unexposed. ER-GI visits were assigned to a town based on the zip code of residence in the emergency room database record. Zip codes were matched to town using information from the US Postal Service. Records with zip codes associated with post-office boxes or from States other than Massachusetts were removed from the analysis. When floods were described as county-wide, or no information was provided about the specific towns affected, all towns in the county were considered exposed. Case and referent periods residing in the town where a flood occurred within the hazard period of interest were considered exposed. A time stratified bi-directional referent selection approach was used. This approach has been shown to control for time-invariant confounders, time trends and seasonal variation in the exposure as well as to produce unbiased results using conditional logistic regression models [21], [22]. Time was stratified into 59 equal periods of 32 days between December 1, 2002 and February 1, 2008 (the control period extended beyond the last case visit of December 31, 2007 and prior to the first case day of January 1, 2003 to allow for control selection before and after case visits). Control periods were matched by day of week with a minimum of two weeks lag or lead between the case to allow for delayed effects of the flood and to minimize overlap with the hazard periods. Thus each case could have one or two control periods. For cases with a single control, the control period could occur before or after the case visit depending on when in the 32-day time period the case visit occurred. By stratifying within a relatively narrow time window we were able to help ensure control for seasonal and temporal effects of flooding and gastrointestinal infections, while allowing a hazard period of up to 14 days.

### Statistical Analysis

We applied a fixed-effect conditional logistic regression model, standard for case-crossover studies [19], [20], [23], and shown to be unbiased when the time-stratified bi-directional case-crossover design is used for control selection [21], [22]. To evaluate whether there were differences in patterns across the state, we conducted separate analyses by county. We also conducted stratified analyses by the following age groups: 0–5; 6–18; 19–64; and over 64 years of age. Results are reported as odds ratios (OR) and associated 95% confidence intervals (CI) and are interpreted as the relative increase in odds of ER-GI visits following a flood. Data management and statistical analyses were conducted using Stata SE Version 12 [24] and conditional logistic regression models were fit using the *xtlogit* command. Attributable fractions and population attributable factions were calculated as described by Hanley [25]. Approximate 95% CIs for population attributable fraction estimates were determined as described by Natarajan *et. al.* [26] and 95% CIs for attributable fractions were estimated by the delta method using the *nlcom* command in Stata SE Version 12. Graphics were produced in R version 11 [27] using the ggplot2 package [28].

## Results

**Figure 1:**
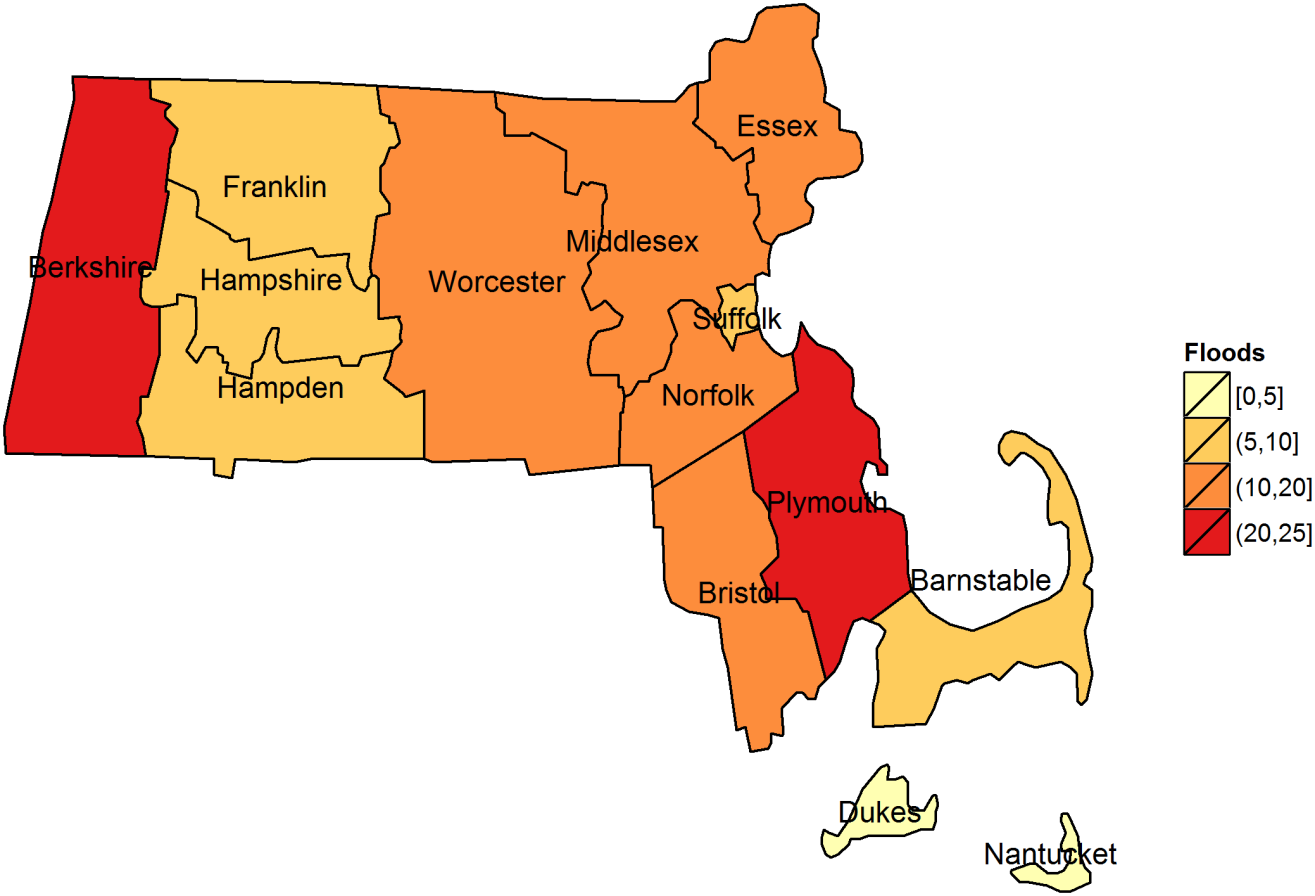
Number of Floods in Massachusetts by county, 2003–2007.

From the last two weeks of 2002 through 2007 there were 129 flood events recorded in Massachusetts in the Storm Events Database. Floods were most frequent in Plymouth (n = 25) and Berkshire (n = 22) counties (Figure 1). Floods were most common in August (21/129, 16%) followed by June (15%) and April (14%), and least common in December (1%), January (2%) and November (5%). No floods were reported in Dukes County during the study period.

During the study period, there were 270,457 ER admissions with at least one ER-GI diagnostic code. In 2003, there were 48,023 admissions and, in 2004, there was a slight decline to 47,265. Thereafter, admissions increased steadily to a maximum of 63,815 in 2007. Including codes 787.01 and 787.03 for non-specific nausea and vomiting increased the number of total ER-GI visits to 464,757 during the study period.

**Figure 2:**
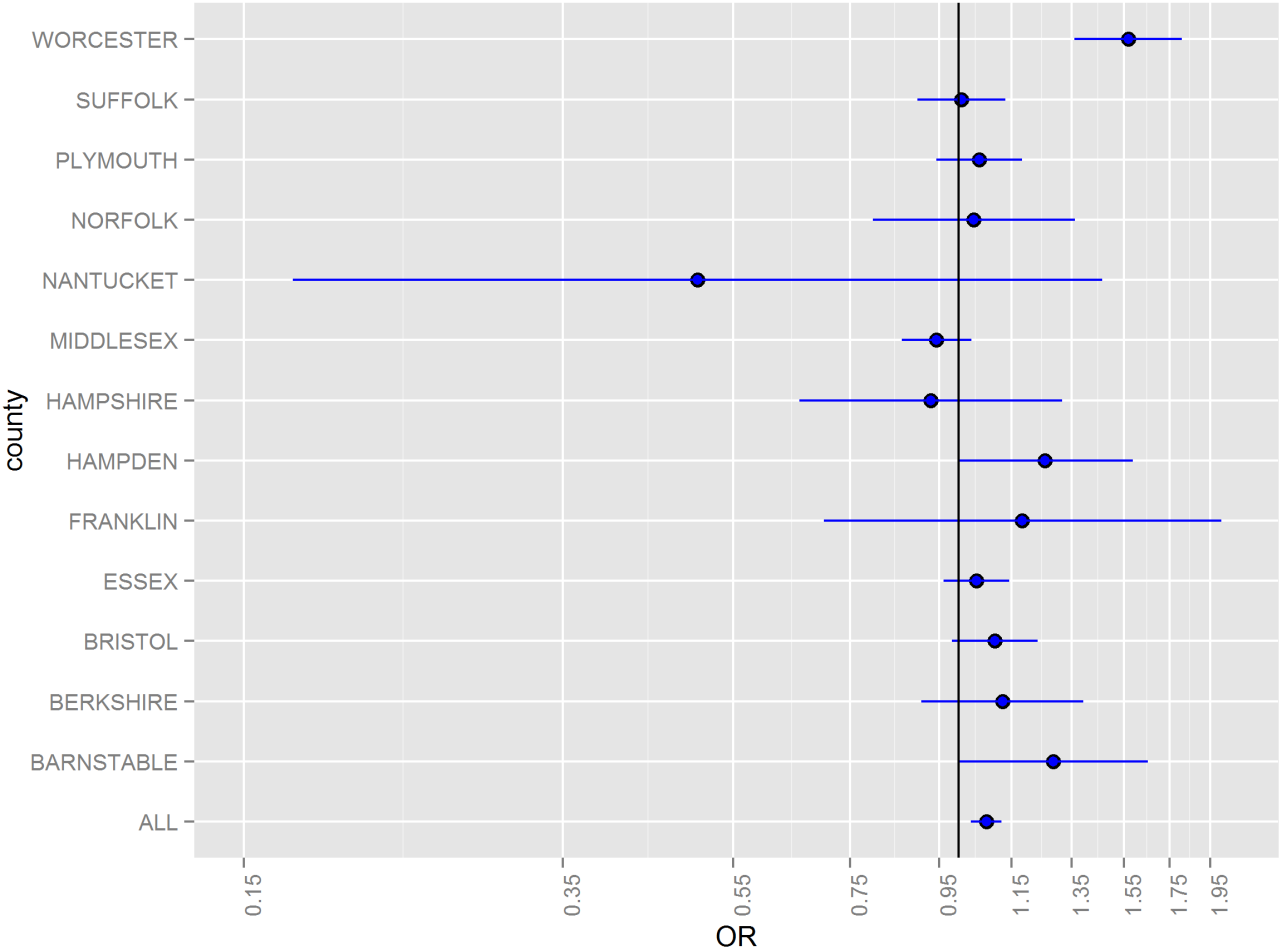
Odds ratios and 95% confidence intervals for emergency room visits for acute gastrointestinal symptoms in the 0–4 days following a flood, 2003–2007.

**Figure 3:**
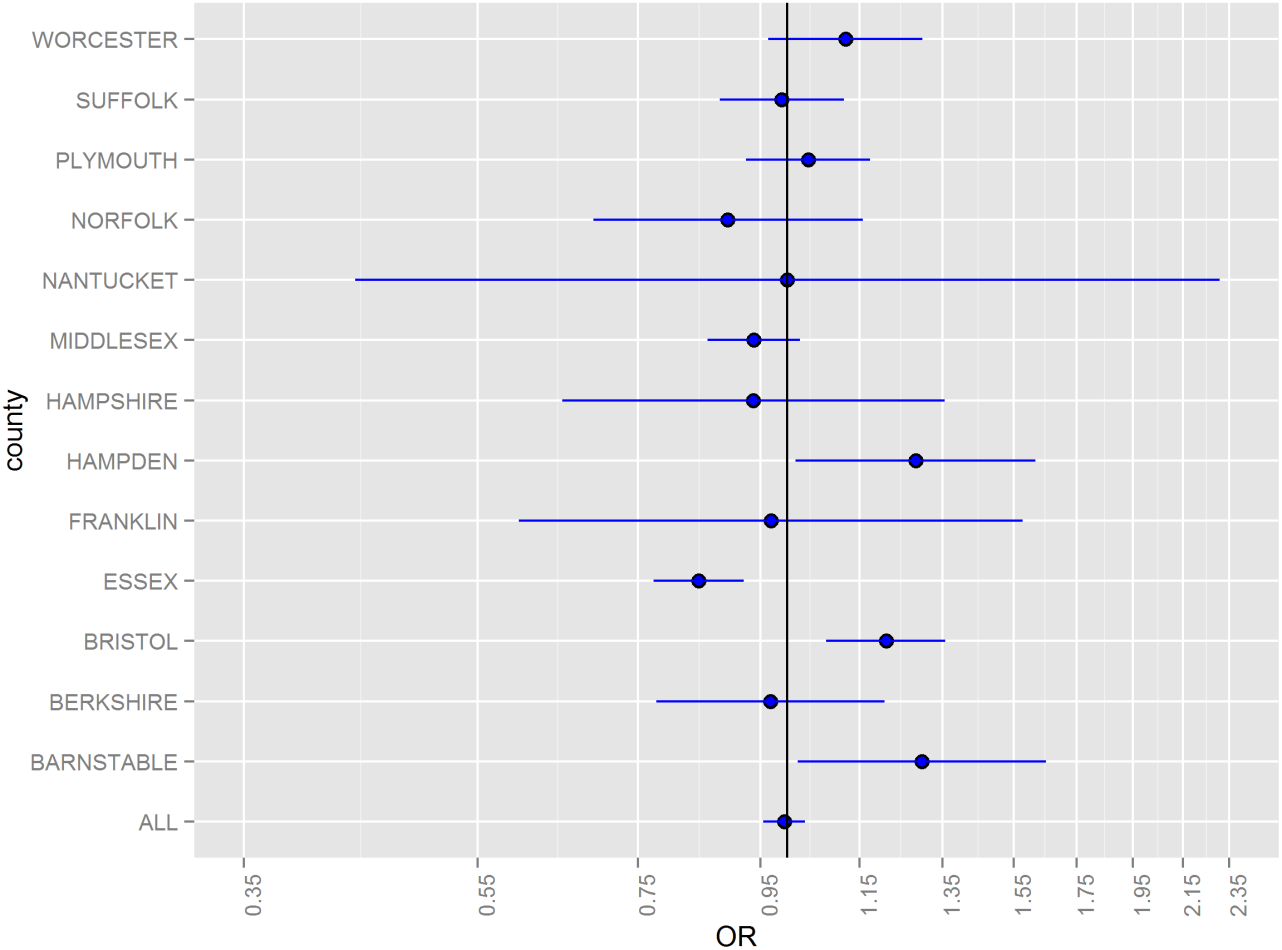
Odds ratios and 95% confidence intervals for emergency room visits for acute gastrointestinal symptoms in the 5–9 days following a flood, 2003–2007.

**Figure 4:**
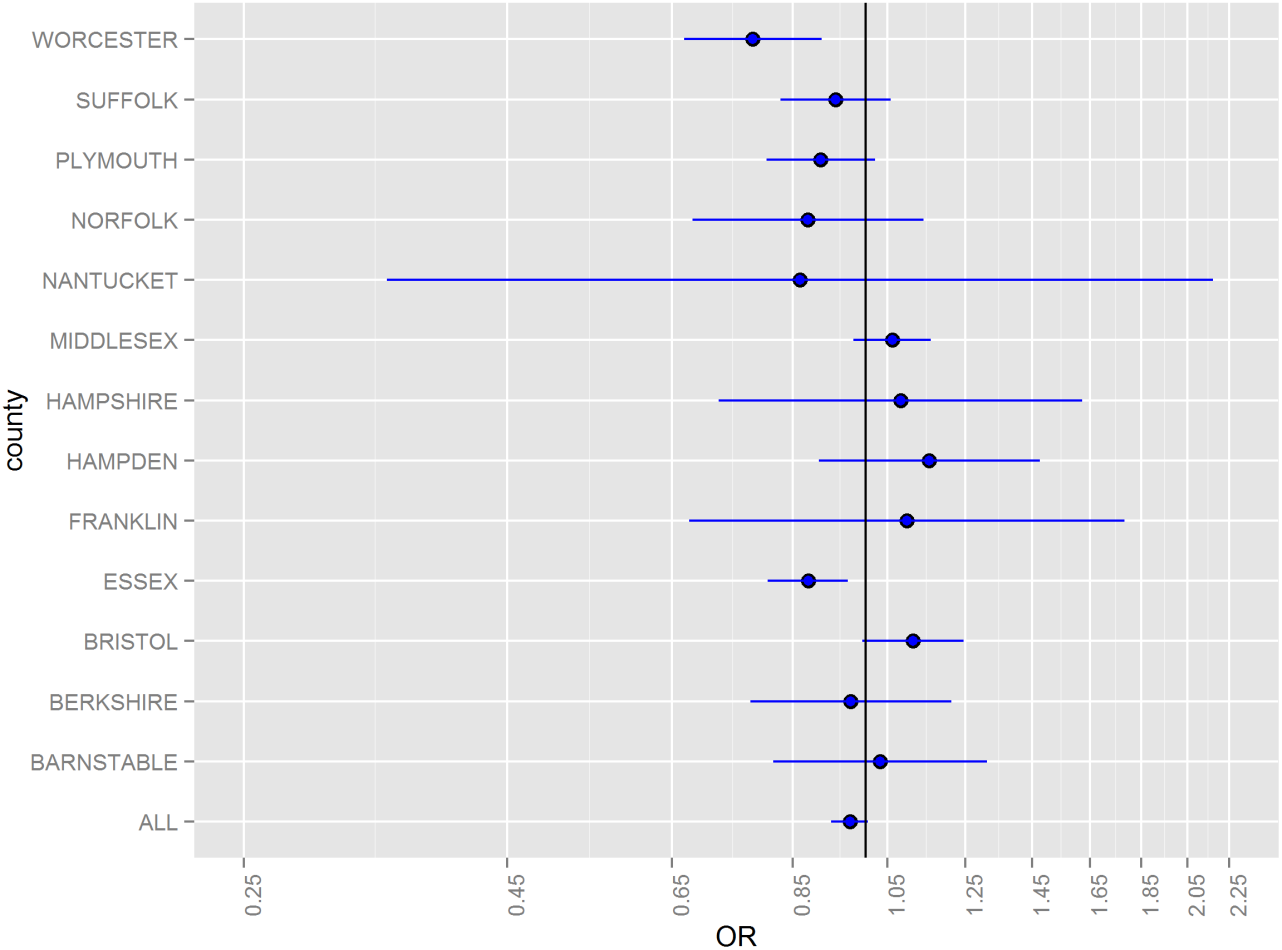
Odds ratios and 95% confidence intervals for emergency room visits for acute gastrointestinal symptoms in the 10–14 days following a flood, 2003–2007.

**Figure 5:**
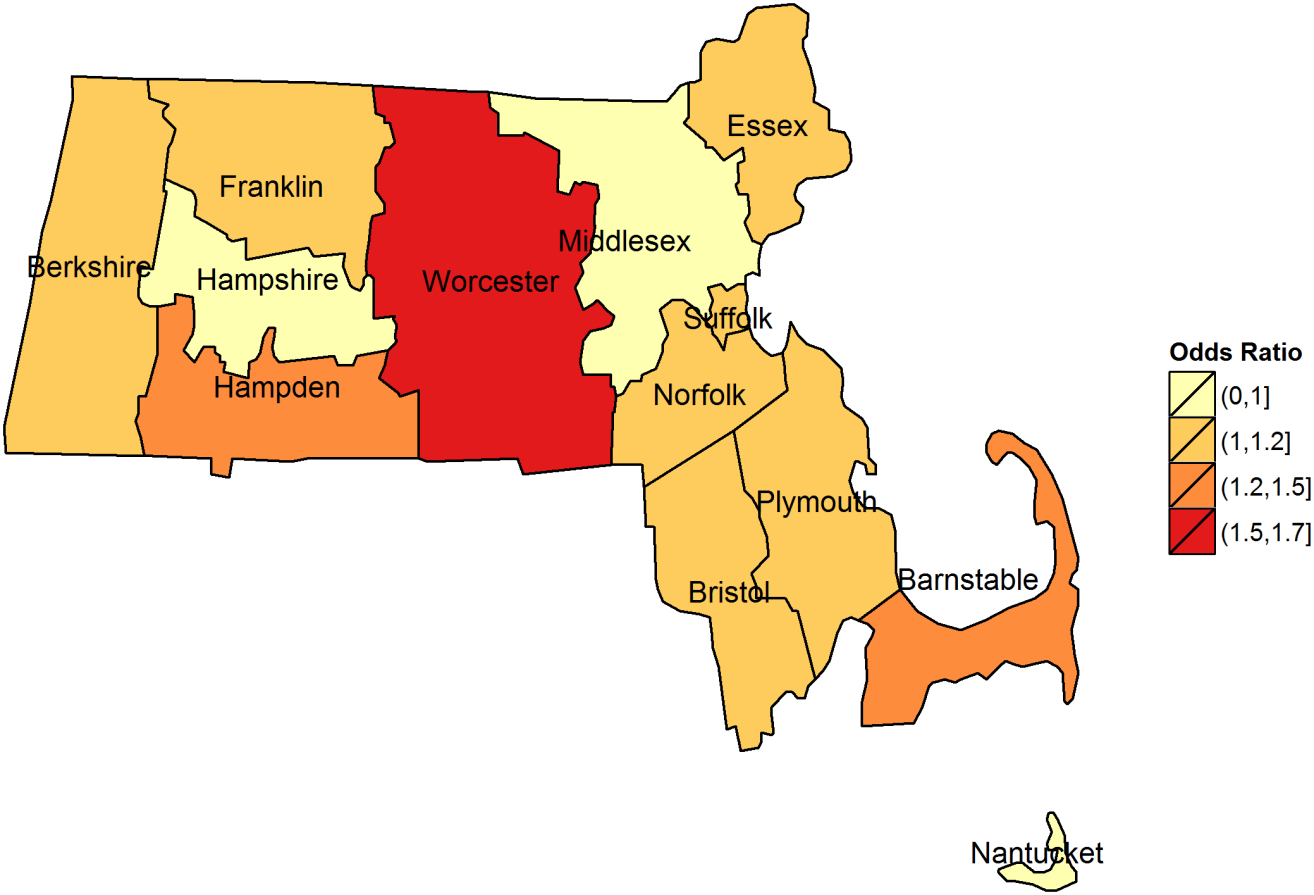
Odds ratios for emergency room visits for acute gastrointestinal symptoms in the 0–4 days following a flood by county, 2003–2007. (No floods occurred in Dukes County, odds ratios were not calculated).

Odds ratios (ORs) for hazard periods 0–4, 5–9, and 10–14 days following floods stratified by county and for all counties combined are illustrated in Figures 2–5. The combined odds ratio for ER-GI visits was 1.08 (95% CI: 1.03–1.12) in the 0–4 days following flooding. In the 5–9 days (OR = 0.995; 95% CI: 0.955–1.04) and the 10–14 days (OR = 0.966; 95% CI: 0.927–1.01) after flooding, there was no evidence of elevated admissions for ER-GI. Results varied by county (Figures 2–4) with the strongest associations observed in Worcester, Hampden, and Barnstable counties for the 0–4 day hazard period (Figure 5).

**Figure 6:**
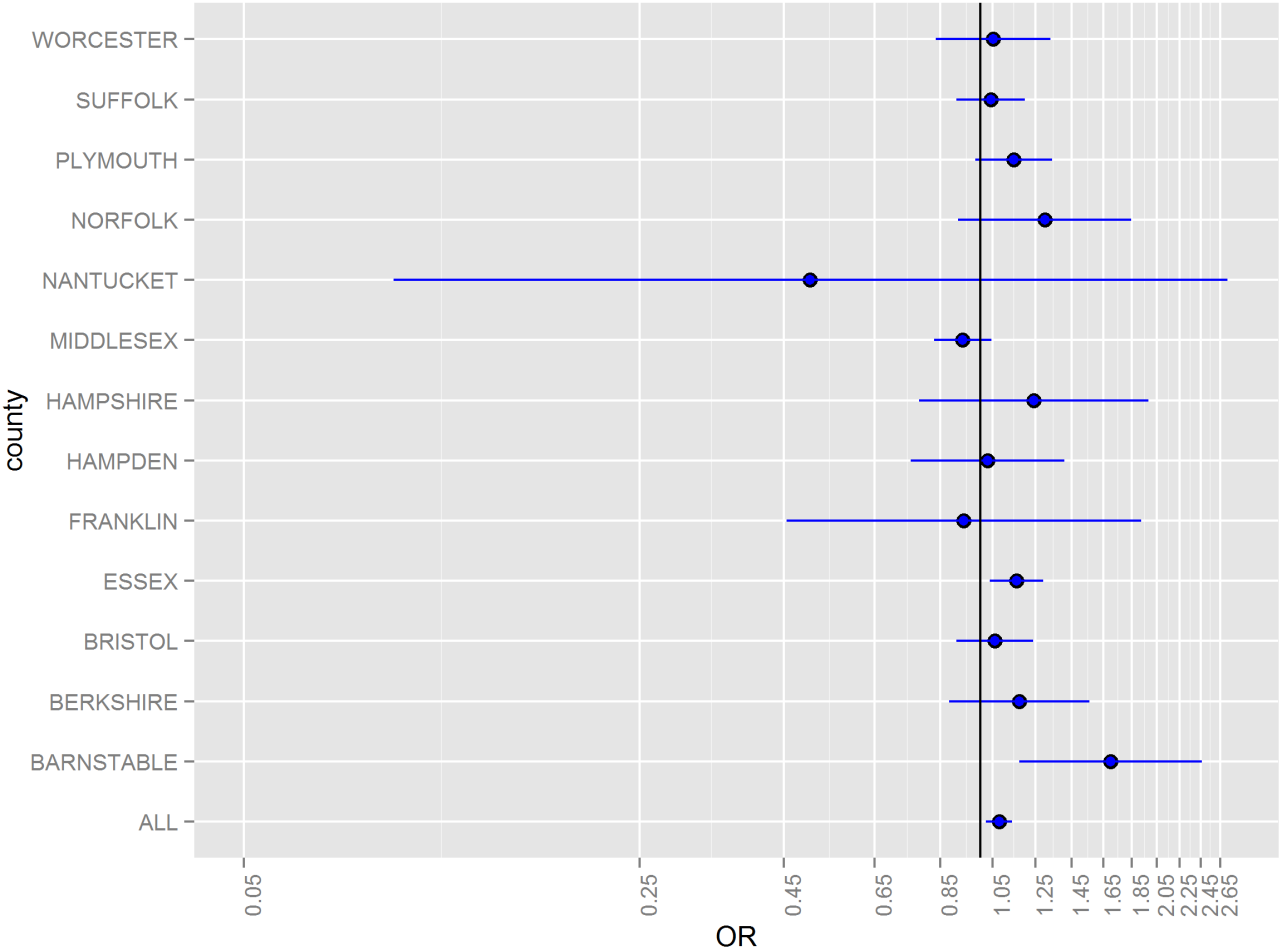
Odds ratios and 95% confidence intervals for emergency room visits for acute gastrointestinal symptoms in the 0–4 days following a flood, 2003–2007. Primary diagnoses only.

**Figure 7:**
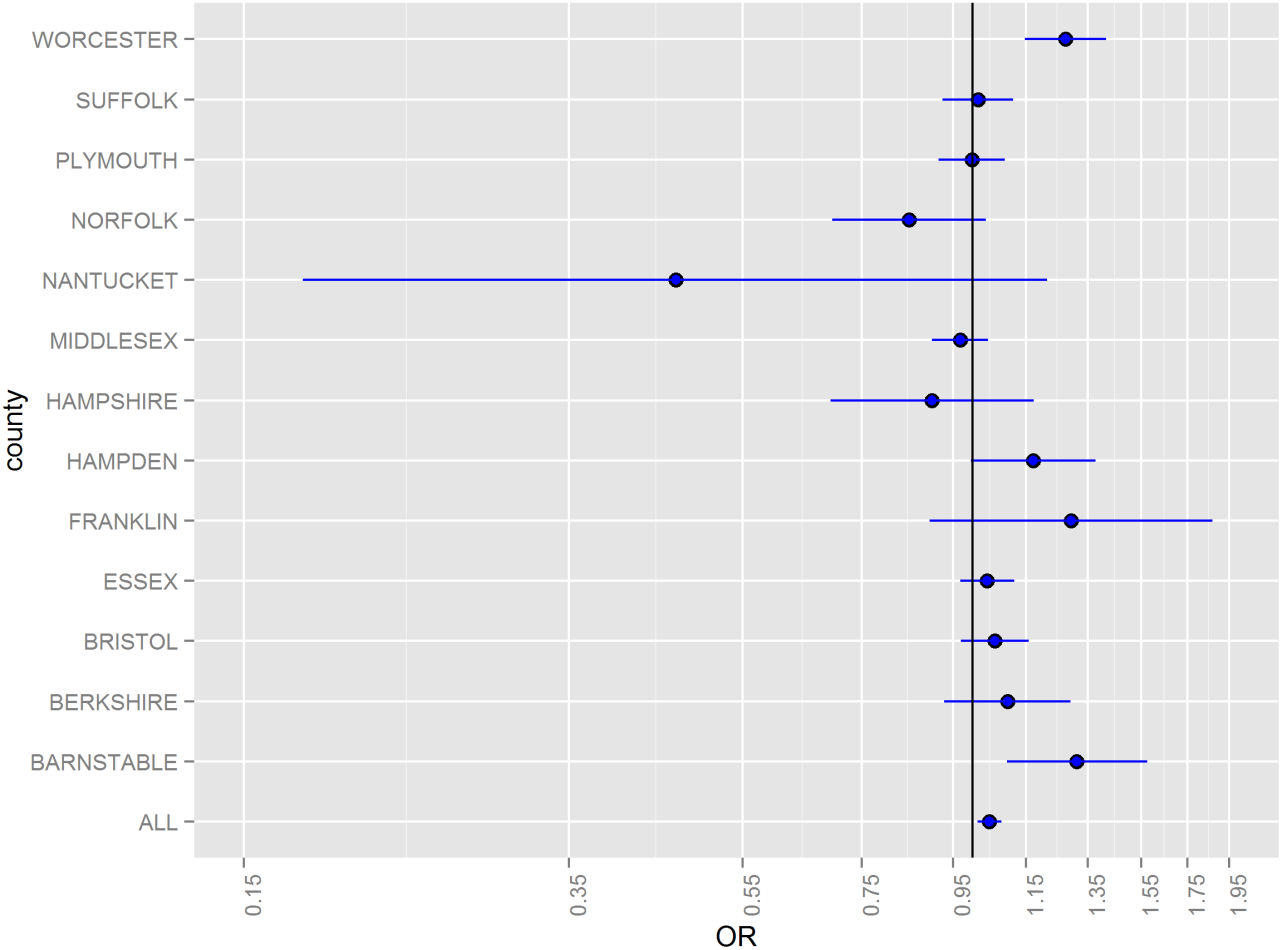
Odds ratios and 95% confidence intervals for emergency room visits for acute gastrointestinal symptoms in the 0–4 days following a flood, 2003–2007. Including non-specific nausea and vomiting.

When we considered primary diagnoses only, the number of cases was reduced to 159,258, but patterns of association with flood events were similar, with ORs combined across county elevated for 0–4 day period (Figure 6) with the same overall association (OR = 1.08; 95% CI = 1.02–1.14) as when all diagnoses were used. Including non-specific symptoms increased the total numbers of visits (465,780), and slightly reduced the point estimate of the overall association with flooding for the 0–4 day hazard period (Figure 7, OR = 1.05; 95% CI: 1.01–1.08). No associations were observed for the 5–9 or 10–14 day periods for either primary diagnoses or ER-GI with non-specific symptoms.

**Figure 8:**
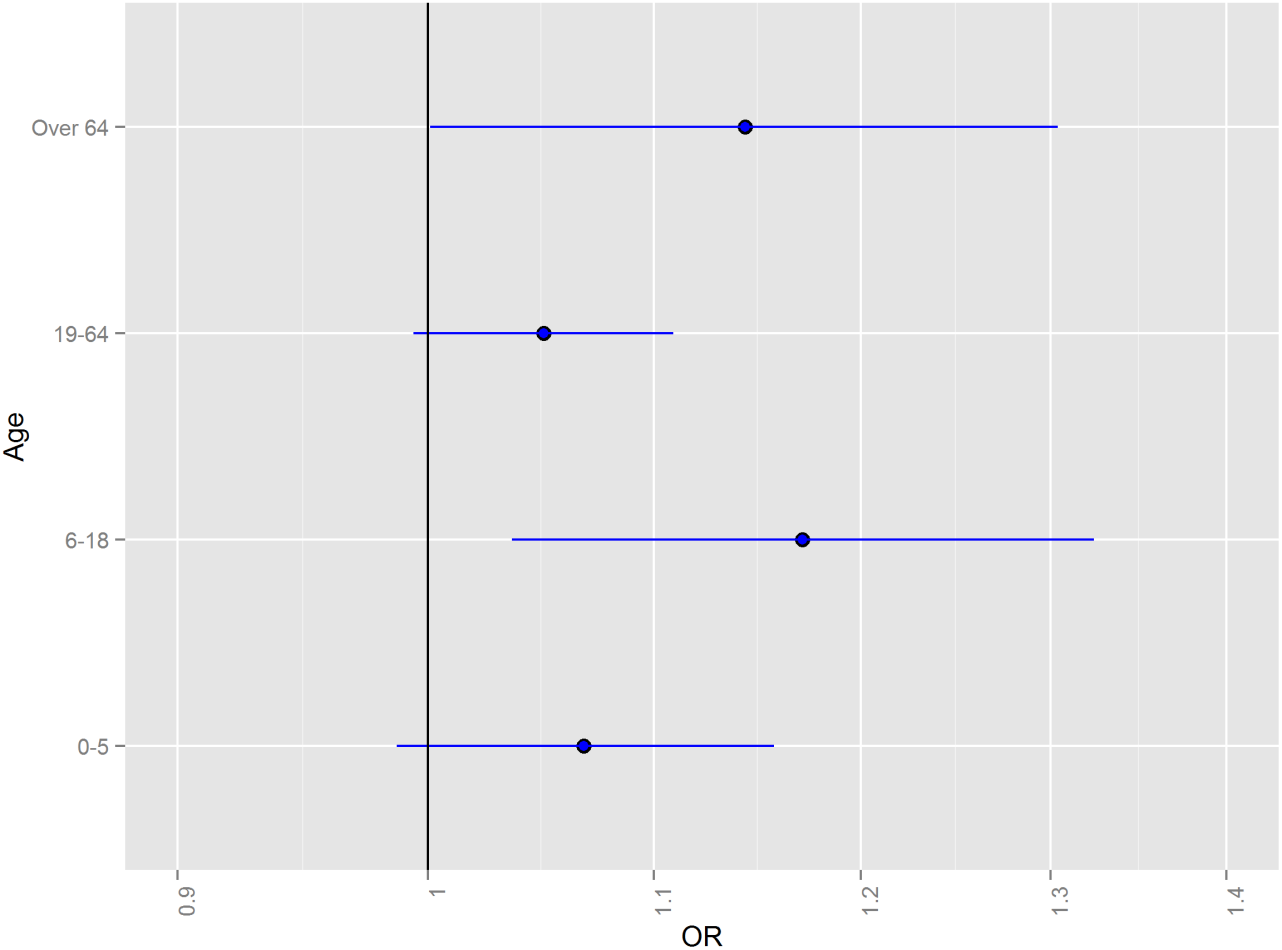
Odds ratios and 95% confidence intervals for emergency room visits for acute gastrointestinal symptoms in the 0–4 days following a flood, 2003–2007 by age group.

Patterns of ER-GI admission associations with flooding were relatively constant across age groups (Figure 8). For the 0–4 day hazard period for all counties, point estimates of the association were slightly higher among the 6–18 age group (OR = 1.17; 95% CI = 1.04–1.32) and the over 64 age group (Figure 8, OR = 1.14; 95% CI = 1.00–1.30), though the 95% confidence bounds of all age-specific estimates overlapped considerably. None of the age groups had elevated risk of ER-GI visits for the 6–9 or 10–14 day periods (data not shown).

### Attributable Fraction Due to Flooding

Based on our results, as many as 7% (95% CI: 3%–11%) of ER-GI visits in the 0–4 days following flooding (attributable fraction = (1.08–1)/1.08 = 0.07) could be attributed to flooding. Considering the fraction of cases which occurred the 0–4 days after flooding (5040/270457 = 0.0186), the fraction ER-GI visits across the state in the entire study period that could be attributed to flooding (population attributable fraction), is 0.13% (95% CI: 0.05%–0.22%), or 1.3 per 1000 (0.07×0.0186 = 0.0013).

## Discussion

Using statewide emergency room visits for gastrointestinal illness, we observed an increased risk for ER-GI visits 0–4 days following flood events in Massachusetts but not in the 5–9 and 10–14 days after floods. By applying a case-crossover design, we controlled for non-varying individual characteristics such as sex and race. In addition, characteristics which are unlikely to vary in the 32 day time-stratified period between the control and case periods such as age, socioeconomic status, and underlying health conditions, were also controlled for in this design. Furthermore, potential season and time trends and day to day variation in care seeking behaviors were controlled through time stratification and by matching by day of week.

A recent case-crossover study examining flooding events in China found a significant increase in reported infectious cases of diarrhea within five days following flooding [29], a result consistent with our findings of a peak in ER-GI visits in the 0–4 days following a flood. Because of its explicit control for non-varying individual factors as well as factors such as seasonality through matching, the case-crossover approach is an efficient epidemiologic study design to study acute health effects associated with transient exposures. Although more commonly applied to the short term effects of air pollution, the design is also well suited to the short term effects associated with severe weather events.

Although the effect we observed was small in magnitude (OR = 1.08 at 0–4 days), assuming this association is valid and the magnitude is approximately correct, during the study period, an estimated 7% of ER-GI visits in the 0–4 days after a flood event could have been attributable to flooding. Moreover, this may be an underestimate of the total impact because GI illnesses that result in ER visits represent a relatively small proportion of the population burden of GI illness [30].

We focused on emergency room visits, which are influenced by factors such as the severity of the illness and access to health care facilities. In spite of the limitations of this measurement of gastrointestinal illness, we observed increased ER-GI visits 0–4 days following a flood. An effect within this hazard period could be consistent with direct or indirect contact with pathogen contaminated water immediately or soon after the flood event. Since the association was only evident in the first four days after flooding, this observation may implicate infections by organisms with relatively short incubation period (e.g., enteric viruses) and direct contact with contaminated waters. Contact with flood waters and flood contaminated items can be fairly frequent. A recent survey in the Netherlands reported that a range from 10% to nearly 70% of those surveyed in areas affected by flooding had some contact with floodwaters [15] and 14% of those surveyed in an Iowa town affected by flooding had at least some exposure to flood waters or flood contaminated items [10].

Variation in the association between flooding and ER-GI admissions was observed across the counties. The reasons for these variations are not addressed by this study, but may be affected by the variability in ER-GI visitations, the nature of the flooding in different counties, the watersheds affected, the population size, and the extent of contamination and impacts on communities and infrastructure. In some cases this variation may also be attributed to few flood events (*e.g.,* Nantucket County). The factors influencing the transmission of gastrointestinal illness following a flood event are complex and depend on numerous factors such as the severity of the flood, the route of transmission (e.g., direct or indirect contact, compromised hygiene or drinking water contamination), the putative pathogenic microorganisms and their incubation period, which can range from less than 1 to over 14 days in some cases, and the underlying immune status of the population.

This assessment has demonstrated the utility of a case-crossover approach and the use of administrative databases in studying acute health effects of weather events. By using a large dataset, we could detect associations between gastrointestinal illness and flooding which may otherwise have gone unnoticed. However, this analysis has several limitations. Only a subset of gastrointestinal illnesses and infections are captured through administrative databases since these illnesses are often not severe enough to require immediate medical attention. Furthermore, we lacked consistent detailed information on the flood events, and were unable to study the impact of flood severity. Additional work could focus on determining the factors influencing the relationship between flooding and gastrointestinal illness by specifically focusing on the nature of watersheds and communities impacted, the nature of the flood, and the potential types of exposure pathways to test specific hypotheses regarding how flooding events increase risk and how these risks could be reduced. Finally, because control periods were represented by specific points during the time window, and we did not use personally identifiable information, we could not confirm that the case did not also visit the ER during the referent period resulting in misclassification of the control. We expect that any resulting misclassification would likely be random with regard to flooding exposure and not result in any systematic bias of the results.

Most evidence for outbreaks of infectious disease following flooding, natural disasters and extreme events is from developing countries that have high endemic levels of disease and inadequate water and sanitation infrastructure. This study provides additional evidence for an association between gastroenteritis and flooding, even in the United States, in the absence of widespread outbreaks. Although this is a preliminary observation and requires additional confirmation, such associations could contribute to a better understanding of the overall impacts of flooding and other related severe weather events resulting from climate change and help inform the public health response following these events.
